# Exploring the Role of Vitamin D in Atherosclerosis and Its Impact on Cardiovascular Events: A Comprehensive Review

**DOI:** 10.7759/cureus.42470

**Published:** 2023-07-26

**Authors:** Shubham Khanolkar, Sajid Hirani, Aditi Mishra, Sauvik Vardhan, Shoyeb Hirani, Roshan Prasad, Mayur Wanjari

**Affiliations:** 1 Medicine, Jawaharlal Nehru Medical College, Datta Meghe Institute of Higher Education and Research, Wardha, IND; 2 Medicine, Mahatma Gandhi Mission (MGM) Medical College and Hospital, Aurangabad, IND; 3 Internal Medicine, Jawaharlal Nehru Medical College, Datta Meghe Institute of Higher Education and Research, Wardha, IND; 4 Research and Development, Jawaharlal Nehru Medical College, Datta Meghe Institute of Higher Education and Research, Wardha, IND

**Keywords:** supplementation, lipid metabolism, endothelial function, immune response, inflammation, cardiovascular events, atherosclerosis, vitamin d

## Abstract

This review explores the role of vitamin D in atherosclerosis and its impact on cardiovascular events. Atherosclerosis, a chronic inflammatory disease characterized by plaque accumulation in arterial walls, is a major contributor to cardiovascular events such as heart attacks and strokes. Vitamin D has emerged as a multifunctional hormone with pleiotropic effects, extending beyond its traditional role in calcium and bone metabolism. Through its anti-inflammatory, immunomodulatory, and antioxidative properties, vitamin D may influence the development and progression of atherosclerosis. The association between vitamin D deficiency and atherosclerosis has been extensively studied. Observational studies consistently report an inverse relationship between vitamin D levels, atherosclerotic risk factors, and markers of subclinical atherosclerosis. Mechanistically, vitamin D exerts anti-inflammatory effects, modulates immune responses, improves endothelial function, and influences lipid metabolism, all of which play critical roles in atherosclerosis development and plaque stability. Furthermore, vitamin D deficiency has been linked to an increased risk of cardiovascular events. Vitamin D influences thrombosis, platelet aggregation, arterial stiffness, blood pressure regulation, and overall vascular health, collectively contributing to cardiovascular event risk. However, the clinical implications of vitamin D for managing atherosclerosis and reducing cardiovascular event risk are still being explored. Randomized controlled trials investigating the cardiovascular benefits of vitamin D supplementation have yielded mixed results, necessitating further research to determine optimal dosages, durations, and patient populations. The review also addresses public health recommendations and future directions. Examining current guidelines, identifying research gaps, and considering public health implications are crucial for translating scientific knowledge into effective interventions. Raising awareness, implementing population-level strategies, and integrating vitamin D assessment into routine clinical practice are key to improving cardiovascular outcomes.

## Introduction and background

Atherosclerosis, a chronic inflammatory disease characterized by plaque accumulation in arterial walls, is a leading cause of cardiovascular events such as heart attacks and strokes. Understanding the underlying mechanisms and risk factors associated with atherosclerosis is crucial for developing effective preventive and therapeutic strategies. In recent years, there has been growing interest in exploring the role of vitamin D in atherosclerosis and its impact on cardiovascular health [1-3].

Vitamin D, traditionally known for its role in calcium and bone metabolism, has emerged as a multifunctional hormone with pleiotropic effects on various body systems. Beyond its classical functions, vitamin D has been found to exert immunomodulatory, anti-inflammatory, and antioxidant properties, suggesting its potential involvement in the pathogenesis of atherosclerosis. Given the high prevalence of vitamin D deficiency worldwide and the alarming rates of cardiovascular events, investigating the role of vitamin D in atherosclerosis has gained significant importance [4,5].

This review article aims to comprehensively explore vitamin D's role in atherosclerosis and its impact on cardiovascular events. By synthesizing the current body of knowledge, we aim to shed light on the mechanisms through which vitamin D influences atherosclerosis development, progression, and associated cardiovascular events. Moreover, we will discuss the potential clinical implications of vitamin D for atherosclerosis management and cardiovascular event prevention. This review aims to provide a valuable resource for researchers, clinicians, and policymakers involved in cardiovascular health, aiming to improve patient outcomes and reduce the burden of atherosclerosis-related diseases.

## Review

Vitamin D and its relevance to atherosclerosis

Overview of Vitamin D and Its Sources

Vitamin D, a fat-soluble vitamin, is crucial in various physiological processes beyond its well-known function in calcium and bone metabolism. Understanding the sources of vitamin D is essential for maintaining optimal levels in the body [2]. Vitamin D can be obtained from both dietary sources and sunlight exposure. Dietary sources of vitamin D include fatty fish like salmon, mackerel, and tuna, as well as fortified dairy products such as milk, yogurt, and cheese. These food sources provide vitamin D3 (cholecalciferol), the form of vitamin D most easily utilized by the body [5].

Sunlight exposure is another important source of vitamin D. When the skin is exposed to sunlight, specifically ultraviolet B (UVB) radiation, a precursor molecule in the skin is converted into cholecalciferol. This inactive form of vitamin D is converted into its active form, calcitriol, through a series of processes in the liver and kidneys. Cutaneous synthesis of vitamin D is considered the primary source for individuals with sufficient sun exposure [6].

However, several factors can influence the efficiency of vitamin D synthesis from sunlight. Geographic location plays a role, as people living in higher latitudes with less direct sunlight may have reduced vitamin D synthesis. Seasonal variations also impact vitamin D production, with reduced sunlight exposure during winter months potentially leading to lower vitamin D levels. Additionally, skin pigmentation affects the amount of UVB radiation absorbed, with darker skin requiring longer sun exposure to produce the same amount of vitamin D as lighter skin. Finally, sunscreen with a high sun protection factor (SPF) can block UVB radiation and hinder vitamin D synthesis [7]. Considering these factors, individuals should strive to obtain vitamin D from dietary sources and sensible sunlight exposure. Supplementation may be necessary for those with limited sun exposure or who are at higher risk of vitamin D deficiency due to specific conditions or lifestyle factors. Consulting with a healthcare professional can help determine the appropriate sources and strategies to maintain adequate vitamin D levels according to individual needs.

Biological Functions and Mechanisms of Vitamin D in the Body

Vitamin D binds to the vitamin D receptor (VDR) expressed in various tissues, including the cardiovascular system. Upon binding, the vitamin D-VDR complex undergoes a conformational change, enabling it to act as a transcription factor. This means that the complex can bind to specific regions of DNA, called vitamin D response elements (VDREs), within target genes, thereby regulating their expression [8]. One well-known role of vitamin D is its involvement in maintaining calcium homeostasis and promoting bone health. In this context, vitamin D facilitates the absorption of calcium and phosphorus from the intestine and regulates their levels in the blood, which is crucial for skeletal development and maintenance [9].

Beyond its classical functions, vitamin D has been implicated in numerous biological processes. It plays a significant role in immune modulation by regulating the differentiation and function of immune cells. For example, vitamin D promotes the differentiation of regulatory T cells (Tregs), which help maintain immune tolerance and suppress excessive inflammation. It also inhibits the differentiation and function of pro-inflammatory immune cells, such as T helper 1 (Th1) and Th17 cells, thereby modulating the immune response [10,11].

Vitamin D is also involved in cell proliferation and differentiation. It regulates the expression of genes associated with cell cycle control, which can influence cellular growth and development. Additionally, vitamin D has been shown to promote the differentiation of various cell types, including cardiomyocytes and vascular smooth muscle cells, which are crucial for cardiovascular health [12]. Furthermore, vitamin D exhibits anti-inflammatory properties. It can suppress the production of pro-inflammatory cytokines such as tumor necrosis factor-alpha (TNF-alpha) and interleukin-6 (IL-6) while enhancing the expression of anti-inflammatory cytokines. This inflammatory response modulation contributes to vitamin D's overall immunomodulatory effects.

Understanding the Association Between Vitamin D Deficiency and Atherosclerosis

It is crucial to understand the link between vitamin D deficiency and atherosclerosis to comprehend its underlying mechanisms and potential therapeutic implications. Observational studies consistently demonstrate an inverse relationship between vitamin D deficiency and atherosclerotic risk factors. Low vitamin D levels are associated with hypertension, dyslipidemia, insulin resistance, and obesity, all of which contribute to the development and progression of atherosclerosis [13]. Moreover, vitamin D deficiency has been linked to markers of subclinical atherosclerosis, such as increased carotid intima-media thickness and coronary artery calcification. These findings suggest that vitamin D plays a significant role in the pathogenesis of atherosclerosis [14].

The precise mechanisms underlying the association between vitamin D deficiency and atherosclerosis are still being investigated. One proposed mechanism is the anti-inflammatory effects of vitamin D. It inhibits the production of pro-inflammatory cytokines. It reduces the activation of immune cells involved in the atherosclerotic process. Vitamin D may attenuate the inflammatory burden within arterial walls by modulating the inflammatory response and inhibiting atherosclerosis development and progression [15]. Additionally, vitamin D may influence endothelial function, oxidative stress, and vascular smooth muscle cell proliferation, critical processes in atherosclerosis. Vitamin D can improve endothelial function by enhancing nitric oxide (NO) production, reducing oxidative stress, and suppressing inflammation. Furthermore, it may regulate the proliferation and migration of vascular smooth muscle cells, contributing to plaque stability.

Mechanisms of vitamin D in atherosclerosis

Anti-inflammatory Properties of Vitamin D and Their Impact on Atherosclerosis

Vitamin D possesses well-established anti-inflammatory properties that have implications for atherosclerosis. When vitamin D levels are insufficient, there is an increased production of pro-inflammatory markers, leading to an imbalance in the inflammatory response. In atherosclerosis, vitamin D exerts its anti-inflammatory effects by suppressing the production of pro-inflammatory cytokines such as TNF-alpha and IL-6. These cytokines are known to contribute significantly to the pathogenesis of atherosclerosis by promoting endothelial dysfunction, facilitating the recruitment of leukocytes to the arterial walls, and facilitating the formation of foam cells within atherosclerotic plaques [16].

Endothelial dysfunction, characterized by impaired endothelial cell function and reduced NO production, is a key early event in atherosclerosis development. Vitamin D's anti-inflammatory properties help mitigate endothelial dysfunction by reducing the levels of pro-inflammatory cytokines that impair endothelial function. By modulating the inflammatory response, vitamin D helps maintain the integrity and functionality of the endothelial lining, which is critical for preventing the initiation and progression of atherosclerosis [17].

Furthermore, vitamin D's anti-inflammatory effects extend to other key processes in atherosclerosis. It inhibits the activation of immune cells, such as monocytes and macrophages, which are central players in atherosclerotic plaque formation and progression. Vitamin D also suppresses the expression of adhesion molecules on endothelial cells, thereby reducing the adhesion and migration of leukocytes into the arterial walls. Additionally, it hampers the transformation of macrophages into foam cells, which are cholesterol-laden cells that contribute to forming fatty plaques [18].

Modulation of the Immune Response and Its Implications for Atherosclerosis Progression

Modulation of the immune response by vitamin D is a critical aspect with profound implications for the progression of atherosclerosis. Vitamin D's immunomodulatory effects influence the delicate balance between pro-inflammatory and anti-inflammatory immune cells. One of the key mechanisms through which vitamin D impacts the immune response is by promoting the differentiation of Tregs. Tregs are a specialized subset of immune cells that play a crucial role in maintaining immune tolerance and preventing excessive inflammation within the arterial walls [19].

By promoting Treg differentiation, vitamin D helps shift the immune response towards an anti-inflammatory phenotype. These Tregs act as suppressors of inflammation, preventing an overactive immune response that could contribute to the progression of atherosclerosis. They play a crucial role in suppressing the activation and function of pro-inflammatory immune cells, such as Th1 and Th17 cells [20].

The Th1 and Th17 cells promote inflammation and contribute to the development and progression of atherosclerosis. Vitamin D inhibits the differentiation and function of these pro-inflammatory immune cells, thereby mitigating the inflammatory burden within the arterial walls. By suppressing the activity of Th1 and Th17 cells, vitamin D helps reduce inflammation and stabilize atherosclerotic plaques [21].

The immunomodulatory effects of vitamin D have significant implications for atherosclerosis progression and plaque stability. The balance between pro-inflammatory and anti-inflammatory immune responses is crucial in determining the extent of inflammation within the arterial walls and the subsequent development and progression of atherosclerosis. By promoting an anti-inflammatory immune environment and suppressing pro-inflammatory immune cells, vitamin D plays a vital role in mitigating inflammation and maintaining plaque stability [22].

Role of Vitamin D in Endothelial Function and Its Effects on Atherosclerosis Development

The role of vitamin D in endothelial function and its effects on atherosclerosis development are crucial aspects to consider in understanding the impact of vitamin D on cardiovascular health. Endothelial dysfunction, characterized by impaired NO bioavailability, increased oxidative stress, and heightened pro-inflammatory signaling, plays a pivotal role in the early stages of atherosclerosis [23].

Vitamin D has been shown to have beneficial effects on endothelial function. It promotes NO production, a key molecule that regulates vascular tone and maintains optimal blood flow. Nitric oxide acts as a vasodilator, helping to relax blood vessels and improve endothelial function. By enhancing NO production, vitamin D helps counteract the negative effects of endothelial dysfunction, thereby supporting the maintenance of healthy blood vessel function [24].

Moreover, vitamin D exerts antioxidant properties, reducing oxidative stress within the endothelial cells. Oxidative stress is a key contributor to endothelial dysfunction, causing damage to the endothelial lining and impairing its ability to regulate blood flow and vascular health. Vitamin D's antioxidant effects help protect endothelial cells from oxidative damage, preserving their functionality and promoting vascular health [25].

Additionally, vitamin D plays a role in suppressing inflammation within the endothelium. Chronic inflammation is a hallmark of endothelial dysfunction and atherosclerosis development. By reducing pro-inflammatory signaling, vitamin D helps alleviate the inflammatory burden on endothelial cells, improving endothelial function and reducing the risk of atherosclerosis progression [26].

Vitamin D regulates endothelial cell function by interacting with the endothelial nitric oxide synthase (eNOS) pathway. Endothelial nitric oxide synthase is an enzyme responsible for producing NO in the endothelium. Vitamin D enhances the expression and activity of eNOS, thus promoting the production of NO and supporting optimal endothelial function. The eNOS pathway is essential for maintaining vascular homeostasis and preventing the development of endothelial dysfunction [27].

Influence of Vitamin D on Lipid Metabolism and Plaque Stability

The influence of vitamin D on lipid metabolism and plaque stability is of significant importance in atherosclerosis. Dyslipidemia, characterized by imbalances in cholesterol levels, particularly elevated low-density lipoprotein cholesterol (LDL-C) and reduced high-density lipoprotein cholesterol (HDL-C), is a major risk factor for the development of atherosclerosis [28].

Vitamin D has been shown to have beneficial effects on lipid metabolism. It promotes the expression of specific genes involved in cholesterol efflux, such as ATP-binding cassette transporter A1 (ABCA1). The ABCA1 is crucial in removing cholesterol from macrophages, which are key players in forming atherosclerotic plaques. By promoting cholesterol efflux, vitamin D inhibits the formation of foam cells, lipid-laden macrophages central to the progression of atherosclerosis. This mechanism contributes to the stability of the plaques, as foam cell accumulation within the arterial wall can lead to plaque rupture and subsequent cardiovascular events [29].

Furthermore, vitamin D can influence the expression and activity of enzymes involved in lipid metabolism, such as hepatic lipase and lipoprotein lipase. These enzymes play important roles in lipoprotein metabolism, affecting the levels of LDL-C and HDL-C. By modulating the expression and activity of these enzymes, vitamin D can potentially regulate lipid profiles and contribute to maintaining a favorable balance between LDL-C and HDL-C, which is essential for preventing the development and progression of atherosclerosis [30].

The effects of vitamin D on lipid metabolism and foam cell formation have important implications for plaque stability. Stable plaques are less prone to rupture and thrombosis, reducing the risk of cardiovascular events such as heart attacks and strokes. By promoting cholesterol efflux and modulating lipid metabolism, vitamin D may contribute to stabilizing atherosclerotic plaques, thereby reducing the risk of cardiovascular events in individuals with atherosclerosis [31].

Vitamin D and cardiovascular events

Exploration of the Potential Links Between Vitamin D Deficiency and Increased Risk of Cardiovascular Events

Numerous observational studies have investigated the association between vitamin D deficiency and an increased risk of cardiovascular events, including myocardial infarction, stroke, and cardiovascular mortality. These studies have consistently reported an inverse relationship between vitamin D levels and cardiovascular event risk. Vitamin D deficiency has been associated with endothelial dysfunction, impaired myocardial contractility, and increased arterial stiffness, all of which contribute to the development and progression of cardiovascular events. Furthermore, vitamin D deficiency may exacerbate other cardiovascular risk factors, such as hypertension, dyslipidemia, and diabetes [16,32-38].

Effects of Vitamin D on Thrombosis, Platelet Aggregation, and Arterial Stiffness

Vitamin D has been implicated in the regulation of hemostasis and thrombosis. Adequate vitamin D levels have been associated with reduced platelet aggregation and improved fibrinolysis, leading to a lower risk of thrombotic events. Vitamin D deficiency, on the other hand, may promote a prothrombotic state and increase the risk of thromboembolic events. Vitamin D deficiency is also linked to increased arterial stiffness, a marker of vascular aging and cardiovascular risk. Arterial stiffness is associated with reduced vascular compliance and impaired endothelial function, predisposing individuals to hypertension and cardiovascular events. Vitamin D supplementation may improve arterial stiffness and reduce the risk of cardiovascular events in individuals with vitamin D deficiency [39].

Impact of Vitamin D on Blood Pressure Regulation and Vascular Health

Hypertension, a major risk factor for cardiovascular events, is influenced by multiple factors, including vascular tone, renal function, and the renin-angiotensin-aldosterone system. Vitamin D has been found to play a role in blood pressure regulation through several mechanisms. It inhibits the renin-angiotensin system, promotes the production of vasodilatory molecules such as NO, and regulates calcium homeostasis in vascular smooth muscle cells. Vitamin D deficiency has been associated with an increased risk of hypertension, whereas vitamin D supplementation has shown potential benefits for lowering blood pressure. Moreover, vitamin D may exert protective effects on vascular health by preserving endothelial function, reducing oxidative stress, and modulating inflammatory pathways, all of which contribute to maintaining vascular integrity and reducing the risk of cardiovascular events [40].

Clinical implications and therapeutic potential

Evaluation of the Potential Clinical Implications of Vitamin D in Managing Atherosclerosis and Reducing Cardiovascular Event Risk

Evaluating the potential clinical implications of vitamin D in managing atherosclerosis and reducing cardiovascular event risk is crucial for understanding the broader implications of vitamin D in cardiovascular health. Healthcare professionals can identify individuals at higher risk for developing cardiovascular events by examining the relationship between vitamin D deficiency and atherosclerosis. This knowledge enables the implementation of targeted preventive strategies to mitigate the risk [41].

Optimizing vitamin D status has several implications for cardiovascular health. Vitamin D has been shown to possess anti-inflammatory properties, thereby reducing inflammation within the arterial walls. This anti-inflammatory effect is particularly important in atherosclerosis, where chronic inflammation is pivotal in plaque development and progression. By reducing inflammation, vitamin D may help prevent the formation of new plaques and stabilize existing plaques, reducing the risk of plaque rupture and subsequent cardiovascular events [42].

Furthermore, vitamin D has a positive impact on endothelial function. Endothelial dysfunction is a critical early event in the development of atherosclerosis. Vitamin D promotes proper vasodilation and regulates blood flow by improving endothelial function, leading to better vascular health. Additionally, vitamin D has been shown to modulate lipid metabolism, helping to maintain a favorable lipid profile. It can enhance the expression of genes involved in cholesterol efflux, reducing the formation of foam cells and the accumulation of cholesterol within the arterial walls. This, in turn, contributes to plaque stability and reduces the risk of cardiovascular events [23].

Addressing vitamin D deficiency and ensuring sufficient vitamin D levels is crucial to managing atherosclerosis and reducing cardiovascular event risk. Healthcare professionals can intervene in the early stages of atherosclerosis by identifying individuals with vitamin D deficiency and implementing appropriate interventions, such as lifestyle modifications and vitamin D supplementation. By optimizing vitamin D status, healthcare professionals can potentially improve cardiovascular outcomes and reduce the burden of cardiovascular disease [35].

Discussion of Vitamin D Supplementation as a Therapeutic Approach and Its Potential Benefits

Vitamin D supplementation has gained attention as a potential therapeutic approach to managing atherosclerosis and reducing cardiovascular event risk. Clinical trials investigating the effects of vitamin D supplementation on cardiovascular outcomes have generated varying results. Some studies have shown promising benefits associated with vitamin D supplementation for cardiovascular health [33]. One potential benefit of vitamin D supplementation is its ability to reduce blood pressure. Several studies have demonstrated that vitamin D supplementation can lead to modest reductions in systolic and diastolic blood pressure, particularly in individuals with hypertension. Lowering blood pressure is important for reducing the risk of cardiovascular events such as heart attacks and strokes [43].

Additionally, vitamin D supplementation has been associated with improvements in lipid profiles. Some studies have reported a decrease in total cholesterol, LDL cholesterol, and triglyceride levels, along with an increase in HDL cholesterol levels, following vitamin D supplementation. These favorable changes in lipid profiles contribute to the overall reduction of cardiovascular risk [44]. Furthermore, vitamin D supplementation has been suggested to have anti-inflammatory effects. Inflammation plays a crucial role in the development and progression of atherosclerosis. Several studies have indicated that vitamin D supplementation can decrease inflammation markers such as C-reactive protein (CRP) and IL-6. These anti-inflammatory properties of vitamin D may contribute to the prevention and management of atherosclerosis [45].

However, it is important to note that not all studies investigating the cardiovascular benefits of vitamin D supplementation have shown consistent results. Some trials have failed to demonstrate significant improvements in cardiovascular outcomes with vitamin D supplementation. Factors such as variations in study design, population characteristics, dosage, and duration of supplementation may contribute to the discrepancies observed [42]. Further research is warranted to fully understand the therapeutic potential of vitamin D supplementation in the context of atherosclerosis and cardiovascular disease. Future studies should focus on elucidating the optimal dosage and duration of supplementation, identifying specific patient populations that may benefit the most, and investigating potential interactions with other cardiovascular therapies.

Consideration of the Optimal Vitamin D Levels for Cardiovascular Health and Prevention of Atherosclerosis

Determining the optimal vitamin D levels for cardiovascular health and prevention of atherosclerosis is a topic of ongoing discussion and research. Current guidelines primarily focus on maintaining serum 25-hydroxyvitamin D levels above 20 ng/mL (50 nmol/L) to prevent bone-related complications. However, regarding cardiovascular health, the ideal range of vitamin D levels is still under debate [46].

Emerging evidence suggests that higher vitamin D levels may be associated with improved cardiovascular outcomes. Some studies suggest maintaining levels above 30 ng/mL (75 nmol/L) could benefit cardiovascular health and atherosclerosis prevention. These higher vitamin D levels may be linked to reduced inflammation, improved endothelial function, better lipid profiles, and a decreased risk of cardiovascular events [47].

On the other hand, other research suggests that levels above 20 ng/mL (50 nmol/L) may be sufficient for cardiovascular health. These studies propose that the relationship between vitamin D and cardiovascular outcomes might follow a U-shaped curve, where both deficiency and excessive levels could be detrimental. Therefore, it is important to strike a balance and avoid both deficiency and excessive supplementation [48]. It is crucial to consider individual patient characteristics, comorbidities, and geographical location when determining the optimal vitamin D levels for cardiovascular health. Factors such as age, ethnicity, sun exposure, dietary intake, kidney function, and medication use can all influence vitamin D status and its impact on cardiovascular health. Personalized approaches to vitamin D supplementation, tailored to individual needs and risk factors, may be necessary to optimize cardiovascular outcomes and prevent atherosclerosis.

Public health recommendations and future directions

Examination of Current Guidelines and Recommendations Regarding Vitamin D Intake and Supplementation

National and international organizations have established guidelines for vitamin D based on preventing bone-related complications, such as rickets and osteoporosis. However, the recommendations addressing cardiovascular health and atherosclerosis prevention are not as well-defined. Evaluating the existing guidelines and their applicability to cardiovascular health can help identify potential gaps and inform future recommendations.

Identification of Research Gaps and Areas for Further Investigation

Prospective studies are warranted to establish the temporal relationship between vitamin D levels and the development of atherosclerosis and cardiovascular events, enabling us to determine causality and assess the potential benefits of early intervention. Well-designed randomized controlled trials are necessary to evaluate the efficacy and safety of vitamin D supplementation in reducing the risk of cardiovascular events while considering diverse patient populations, dosages, and treatment durations to provide robust evidence. Additionally, mechanistic studies are essential to uncovering the specific cellular and molecular pathways through which vitamin D influences atherosclerosis development and cardiovascular health, offering insights into potential therapeutic targets. Finally, research should focus on establishing the optimal range of vitamin D levels for cardiovascular health and atherosclerosis prevention, which can help refine recommendations and guide clinical practice. By addressing these research areas, we can enhance our knowledge and pave the way for more targeted interventions and improved patient outcomes in vitamin D and cardiovascular health.

Implications for Public Health Interventions and Potential Future Directions

Public health campaigns and education programs can raise awareness about the importance of maintaining adequate vitamin D levels and highlight the potential benefits of healthy lifestyle behaviors, including sun exposure and dietary sources of vitamin D. Additionally, future research should explore the feasibility and effectiveness of population-level strategies, such as fortification programs or targeted supplementation, in improving vitamin D status and reducing cardiovascular event risk.

Furthermore, integrating vitamin D assessment into routine clinical practice and cardiovascular risk assessments can enhance risk stratification and guide personalized preventive strategies. Collaborative efforts between researchers, clinicians, and policymakers are crucial to identifying effective public health interventions, bridging research gaps, and promoting evidence-based recommendations for optimizing vitamin D status in the context of atherosclerosis and cardiovascular health.

## Conclusions

The role of vitamin D in atherosclerosis and its impact on cardiovascular events are of great significance. Through this review, we have highlighted the potential mechanisms linking vitamin D deficiency to the development and progression of atherosclerosis and its associations with cardiovascular event risk. The evidence suggests that maintaining adequate vitamin D levels may benefit inflammation, immune response, endothelial function, lipid metabolism, and vascular health, all of which play crucial roles in atherosclerosis and cardiovascular disease. Recognizing the importance of vitamin D for cardiovascular health is essential for developing targeted preventive and therapeutic strategies. However, further research is needed to establish causality, determine optimal vitamin D levels, and assess the clinical efficacy of vitamin D supplementation. Public health should also focus on raising awareness, implementing population-level interventions, and integrating vitamin D assessment into routine clinical practice to improve cardiovascular outcomes. By advancing our understanding of the role of vitamin D in atherosclerosis and cardiovascular events, we can strive towards reducing the burden of cardiovascular disease and improving the overall cardiovascular health of individuals.
